# Comprehensive DFT study of 3-(2-furyl)-1 H-pyrazole-5-carboxylic acid: structural, spectroscopic, and electronic properties with optoelectronic implications

**DOI:** 10.1038/s41598-025-05803-6

**Published:** 2025-07-01

**Authors:** Mücahit Yılmaz

**Affiliations:** Department of Physics, Faculty of Science, Firat University, 23119 Elazig, Türkiye

**Keywords:** 3-(2-furyl)-1H-pyrazole-5-carboxylic acid, Quantum chemical calculations, DFT, MEP, NMR, FT-IR, Atomic and molecular physics, Chemical physics

## Abstract

In this study, the structural and electronic properties of the compound 3-(2-furyl)-1 H-pyrazole-5-carboxylic acid have been theoretically investigated by Density Functional Theory (DFT). The most stable geometry of the molecule was optimized and vibrational frequencies were calculated at the B3LYP/6-31G(d) level. Geometry optimization results indicate that the molecule adopts a planar conformation, meaning all constituent atoms lie within the same geometrical plane. This confirms that the atomic arrangement is not spatially random or dispersed in three dimensions, but instead exhibits a well-defined two-dimensional planar geometry. In addition to geometric optimization, quantum chemical calculations show that the molecule has high electronic stability and low reactivity, and also reveal its potential for optoelectronic applications with strong absorption properties in the UV. These analyses contribute to the structural confirmation of the compound and provide important information on the chemical reactivity of the functional sites. The results obtained show that the compound has significant potential for optoelectronic, pharmaceutical and sensor applications.

## Introduction

Heteroaromatic compounds, with their conjugated systems and functional groups, have a wide application potential in various pharmaceutical, pharmacological, electronic applications, biological and material science fields^1^. Compounds incorporating furan and pyrazole moieties are of particular interest owing to their diverse biological activities and electronic characteristics^2,3^. Such compounds are well known for their anti-inflammatory, antimicrobial and anticancer activities and are also used in sensor technologies and photonic devices due to their electronic properties. In order to predict the physicochemical properties of such complex molecules without experimental data, computational methods such as DFT play a critical role^4,5^. By determining the stable structure of the molecule with the lowest energy, the quantum chemical properties of the molecule are analyzed. B3LYP is known for successfully predicting structural and spectral properties of organic molecules by combining Becke’s three-parameter exchange functional with the Lee-Yang-Parr correlation functional^6–8^.

There are various studies on the synthesis, spectroscopic characterisation, antimicrobial and anti-inflammatory biological activities of 3-(2-furyl)-1 H-pyrazole-5-carboxylic acid and similar derivatives^9–12^. Furthermore, modification of these compounds with different functional groups has been studied with the aim of improving their interactions with biological targets and pharmacokinetic properties^13^. Although several studies have focused on the synthesis and biological activities of 3-(2-furyl)-1 H-pyrazole-5-carboxylic acid and its derivatives^9–13^, a comprehensive quantum chemical analysis covering the structural, spectroscopic, and electronic properties of this compound has not yet been adequately presented. While existing research primarily emphasizes experimental bioactivity or synthetic strategies, the present study offers a systematic DFT-based characterization that includes geometry optimization, vibrational and magnetic spectral simulations (FT-IR and NMR), electronic structure analysis (HOMO–LUMO and DOS), UV–Vis transition assignments obtained via TD-DFT, and molecular electrostatic potential (MEP) surface mapping. Through this multifaceted theoretical approach, the reactivity, stability, and potential suitability of the compound for optoelectronic and sensor applications are evaluated in greater detail.

The 3-(2-furyl)-1 H-pyrazole-5-carboxylic acid molecule is of special importance as it has conjugated π-systems and is open to various molecular interactions with its functional groups. In this study, the quantum chemical properties of this compound have been extensively investigated by DFT. It is aimed to provide information about the reactivity and potential application areas of the molecule by evaluating the electronic structure, spectroscopic behavior and molecular electrostatic properties. The theoretical data obtained provide information about the electronic structure, reactivity centres and optical properties of the molecule and are thought to provide a basis for experimental research.

## Methods

All quantum chemical calculations were performed using the Gaussian 09 software package (version AS64L-G09RevD.01)^14,15^. The initial molecular geometry was fully optimized using the hybrid density functional B3LYP (Becke’s three-parameter exchange functional combined with the Lee-Yang-Parr correlation functional) in conjunction with the Pople-style split-valence double-zeta basis set, extended by polarization functions on heavy atoms, 6-31G(d). To confirm the stability of the optimized structure, vibrational frequency analysis was performed at the same level of theory, ensuring the absence of imaginary frequencies and thus verifying that the obtained structure corresponds to a true local minimum on the potential energy surface. Subsequent electronic structure analyses were performed on the optimized geometry. The energies of the frontier molecular orbitals, namely The Highest Occupied Molecular Orbital (HOMO) and The Lowest Unoccupied Molecular Orbital (LUMO), were calculated and the energy gap (ΔE) was determined as an indicator of the electronic stability and reactivity of the molecule. Fourier Transform Infrared Spectroscopy (FT-IR) and Nuclear Magnetic Resonance (NMR) spectra were simulated to characterize the vibrational and magnetic properties, respectively. Time-dependent density functional theory (TD-DFT) calculations at the B3LYP/6-31G(d) level were performed to explore the electronic excitation properties of the system. The resulting UV-Vis absorption spectrum provided insight into the optical behavior of the molecule. The Density of States (DOS) spectrum was generated to show the energy resolved distribution of electronic states, highlighting contributions from different molecular orbitals. In addition, a molecular electrostatic potential (MEP) map was constructed to visualize the spatial distribution of electrostatic potential across the molecular surface. This map facilitates the identification of electron-rich and electron-deficient regions, providing valuable information on potential reactive sites. All calculated spectra and molecular surface maps were graphically displayed, interpreted in detail and integrated into the discussion for a comprehensive analysis of the physicochemical properties of the compound.

### Result and discussion

#### Geometry optimization

The most stable geometric configuration of the 3-(2-furyl)-1 H-pyrazole-5-carboxylic acid molecule was determined by full geometry optimization using the Gaussian 09 software suite (version AS64L-G09RevD.01) within the framework of DFT. The B3LYP hybrid functional was used in conjunction with the 6-31G(d) basis set to accurately model the electronic structure and molecular geometry. The optimized molecular structure revealed that the constituent atoms are arranged in a near-planar conformation, rather than adopting a random three-dimensional spatial distribution. This planar geometry is stabilized by intramolecular non-covalent interactions. A key stabilizing feature of the optimized structure is the formation of an intramolecular hydrogen bond between the N-H proton of the pyrazole moiety and the carbonyl oxygen of the carboxylic acid group. This interaction contributes significantly to the conformational rigidity and energetic stability of the molecule. The absence of any imaginary vibrational frequencies in the harmonic vibrational frequency analysis confirms that the optimized structure corresponds to a true local minimum on the potential energy surface.

**Fig. 1:**
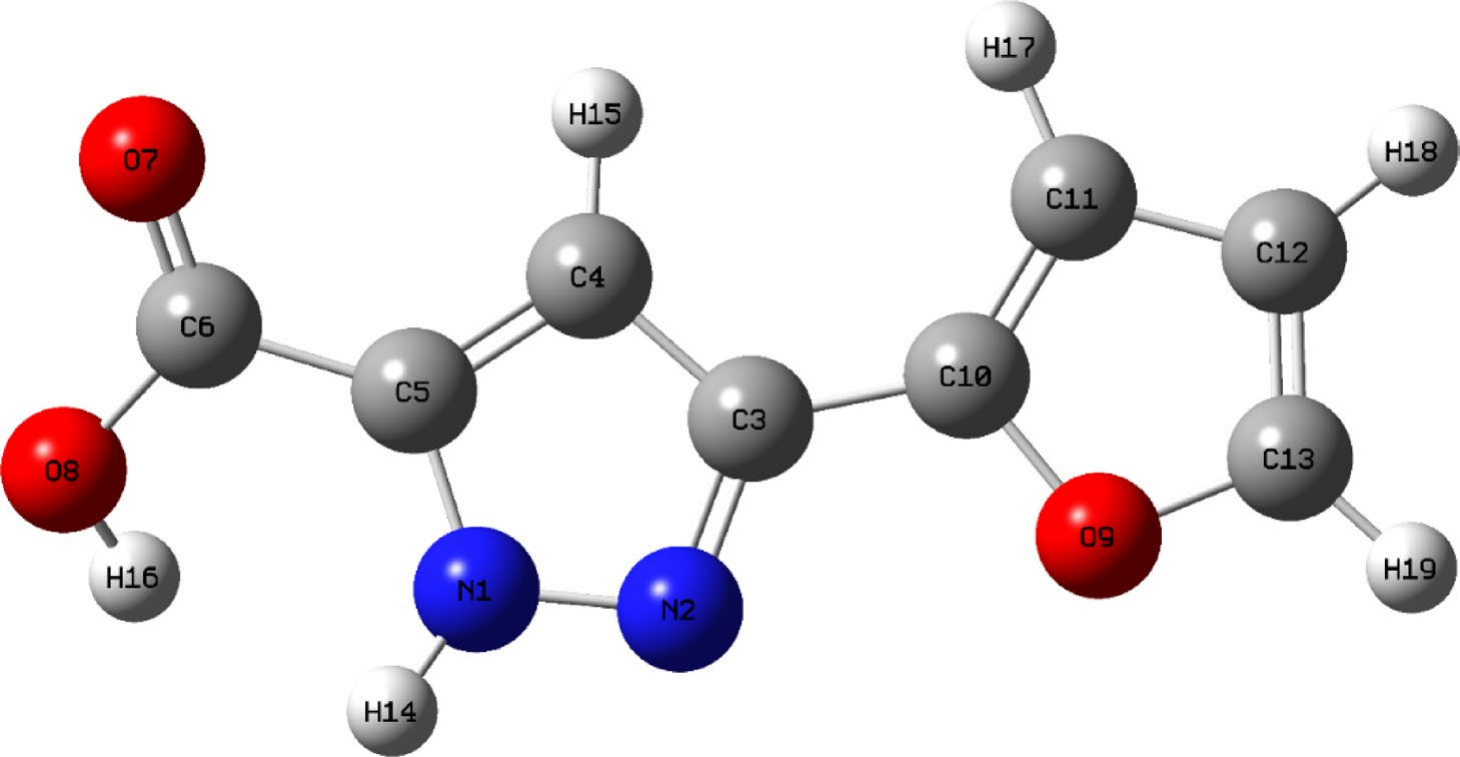
Optimized structure of 3-(2-furyl)-1 H-pyrazole-5-carboxylic acid molecule.

The molecular backbone consists of three distinct functional domains: a pyrazole ring, a furyl group and a carboxylic acid substituent^16^. The pyrazole ring is composed of atoms C3, C4, C5, N1 and N2, with the furyl moiety covalently attached to the C3 position. The aromaticity of the pyrazole ring is maintained by delocalization between N1 and N2. A carboxylic acid group is attached to the C5 position, consisting of the carbon atom C6 attached to a carbonyl oxygen atom (O7) and a hydroxyl group (O8-H16). The carbonyl group actively participates in π-electron delocalization with the conjugated system, enhancing the planarity and resonance stabilization of the molecule. In addition, the hydroxyl hydrogen of the carboxylic acid (H16) is favorably positioned to interact via intramolecular hydrogen bonding with the adjacent N1 atom on the pyrazole ring, further enhancing structural stability. The furyl group, linked via the C3 atom, is almost coplanar with the pyrazole ring, promoting extended conjugation across the molecular backbone. Overall, the structural integrity and planarity of the molecule is maintained by the synergistic effects of resonance delocalization and intramolecular hydrogen bonding, as shown in Fig. 1.

## Band gap energy (BG)

**Fig. 2:**
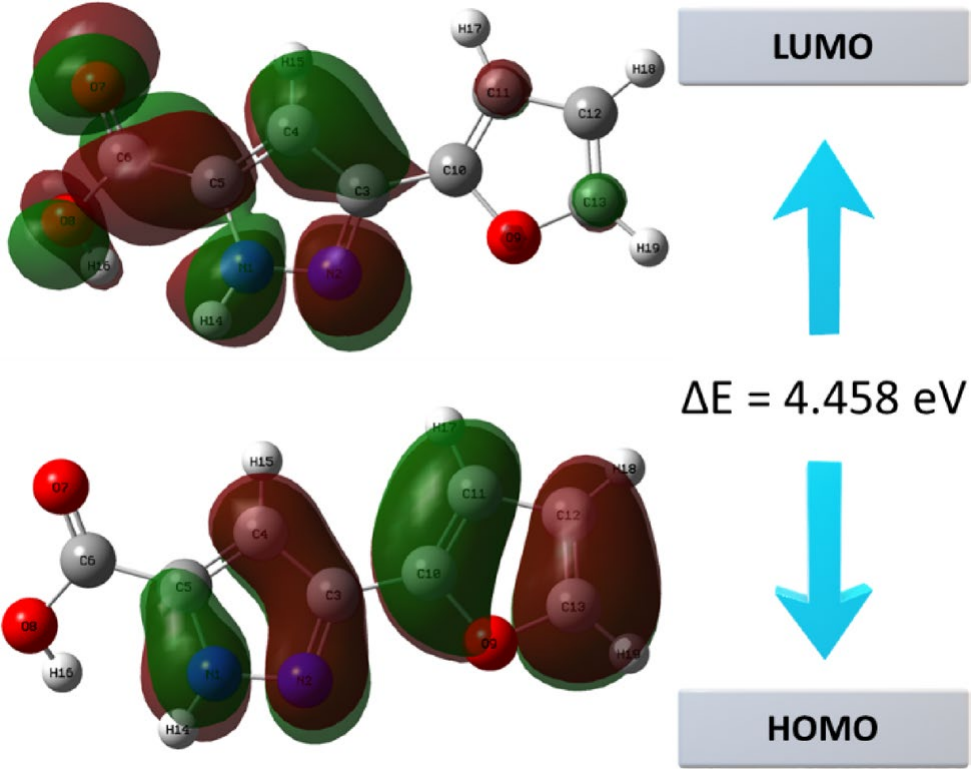
The molecular orbital arrangement and energy state diagram of 3-(2-furyl)-1 H-pyrazole-5-carboxylic acid molecule.

HOMO and LUMO orbitals play a critical role in elucidating key molecular properties such as chemical reactivity, electronic stability, electronic transition behavior, and photoexcitation potential^17,18^. The ΔE energy gap between these orbitals directly influences global reactivity descriptors such as molecular softness, hardness, and polarizability^19^. HOMO and LUMO energy levels, which are critical for determining the electronic transition characteristics and reactivity tendencies of the molecule, were calculated as -5.907 eV and − 1.449 eV, respectively. HOMO and LUMO orbital distributions of the 3-(2-furyl)-1 H-pyrazole-5-carboxylic acid molecule are presented in Fig. 2.

HOMO-LUMO energy gap of the molecule was calculated to be approximately 4.458 eV. This relatively large energy separation suggests that the molecule possesses high electronic stability but may exhibit reactive behavior under specific conditions. The wide band gap also implies a greater difficulty in electron transition from HOMO to the LUMO, thereby indicating low chemical reactivity and high thermodynamic stability.

As shown in the lower panel of Fig. 2, HOMO orbital is predominantly localized in the aromatic systems rich in π-electron density, particularly around the N1 and N2 nitrogen atoms of the pyrazole ring, the C4–C5 double bond, and to a lesser extent, oriented toward the carboxylic acid group. This distribution reveals that the HOMO is concentrated in nucleophilic centers-regions of high electron density. The lone pairs on the nitrogen atoms and their interactions with the conjugated π-system significantly contribute to HOMO orbital.

Conversely, the LUMO orbital, depicted in the upper panel of Fig. 2, is mainly localized over the π* (antibonding) orbitals of the furan ring, the C10–C13 carbon chain, around the C = O group, and especially on the O7 atom. This distribution indicates the electrophilic (electron-deficient) regions of the molecule. Analysis of the orbital shape and phase structure confirms the π-antibonding character of LUMO, which is arranged to resonate with electron-rich regions of the aromatic framework. While the HOMO is primarily localized on the pyrazole ring, LUMO is centered on the furyl moiety and the carboxylic acid group. This spatial separation suggests that these regions are the most probable sites for accepting incoming electrons, implying that they are likely to be targeted by nucleophilic agents.

## Vibrational spectroscopic analysis spectrum

**Fig. 3:**
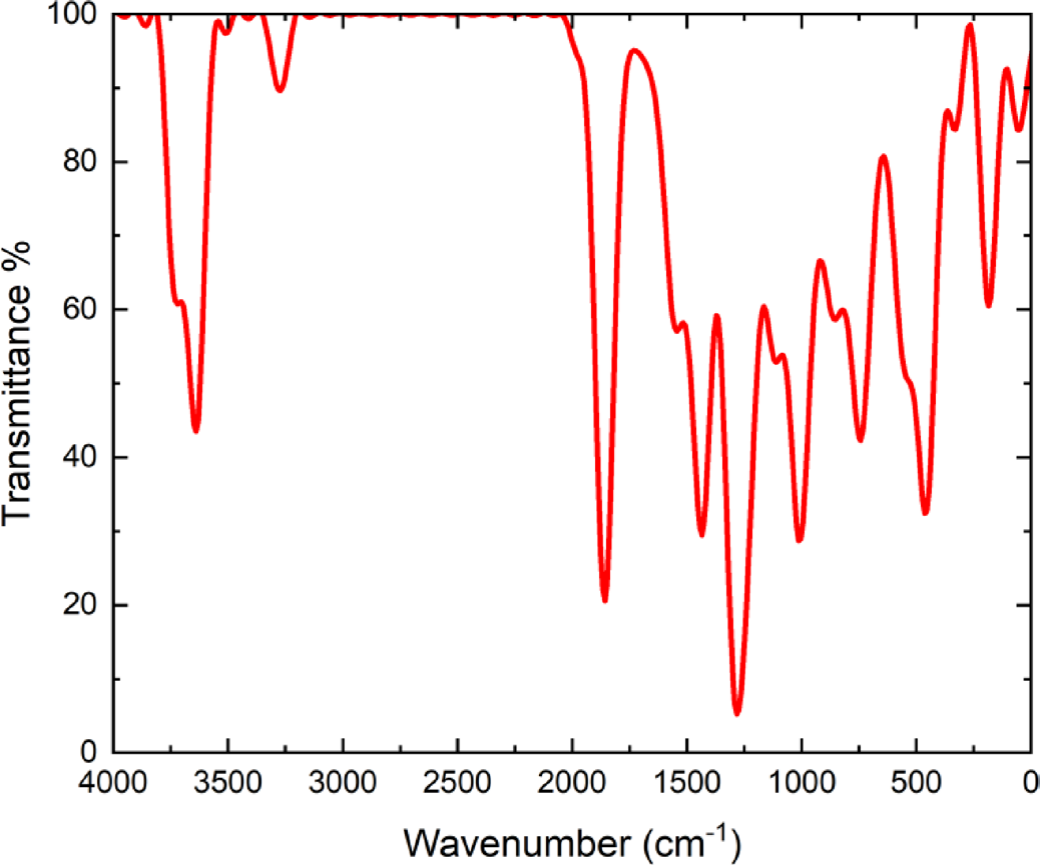
FT-IR spectrum of 3-(2-furyl)-1 H-pyrazole-5-carboxylic acid molecule.

FT-IR is a powerful spectroscopic technique that provides insights into the chemical structure of molecules by analyzing their characteristic vibrational modes. This technique operates by passing infrared radiation through a molecule and measuring the energy absorbed at specific frequencies^20,21^. Each molecule possesses a unique vibrational spectrum, which provides valuable information about the types of bonds present and the influence of their surrounding environment^22^. The FT-IR spectrum of the 3-(2-furyl)-1 H-pyrazole-5-carboxylic acid compound is presented in Fig. 3. In the spectrum, characteristic vibrational bands corresponding to the functional groups of the compound were recorded within the wavenumber range of 4000 –400 cm^− 1^. The vertical axis represents percent transmittance, while the horizontal axis indicates the wavenumber in cm⁻¹.

The broad band observed in the ~ 3400 –3200 cm^− 1^ region of the spectrum is attributed to the O-H stretching vibration of the carboxylic acid group. This strong absorption band is considered to be broad and diffuse due to the presence of hydrogen bonding. The weak to moderate peaks observed between 3100 and 3000 cm^− 1^ correspond to C–H stretching vibrations in aromatic rings, indicating the presence of hydrogen atoms in both the furyl and pyrazole rings of the molecule. A sharp peak around ~ 1700 cm^− 1^ represents the C = O stretching vibration of the carboxylic acid group. The high frequency and strong intensity of this band suggest that the carbonyl group resides in an electron-rich environment. Multiple absorption bands in the ~ 1600 –1500 cm^− 1^ region are associated with C = C and C = N stretching vibrations, with the C = N bond in the pyrazole ring being particularly characteristic in this wavenumber range. The medium to strong bands observed between ~ 1300 –1000 cm^− 1^ correspond mainly to C-O (carboxyl) and C-N (pyrazole ring) stretching vibrations, with potential contributions from C-H bending vibrations in the same region. Weak bands around ~ 800 –600 cm^− 1^ are related to out-of-plane deformations of the aromatic rings.

The characteristic bands observed in the FT-IR spectrum are consistent with the structural integrity of the 3-(2-furyl)-1 H-pyrazole-5-carboxylic acid compound, confirming the presence of the carboxylic acid group, aromatic systems (furyl and pyrazole rings), and nitrogen- and oxygen-containing functional groups. When supported by theoretical calculations, the spectral analysis provides a detailed understanding of the vibrational behavior of the compound and plays a crucial role in verifying the molecular structure.

## Nuclear magnetic resonance spectroscopy (NMR)

**Fig. 4:**
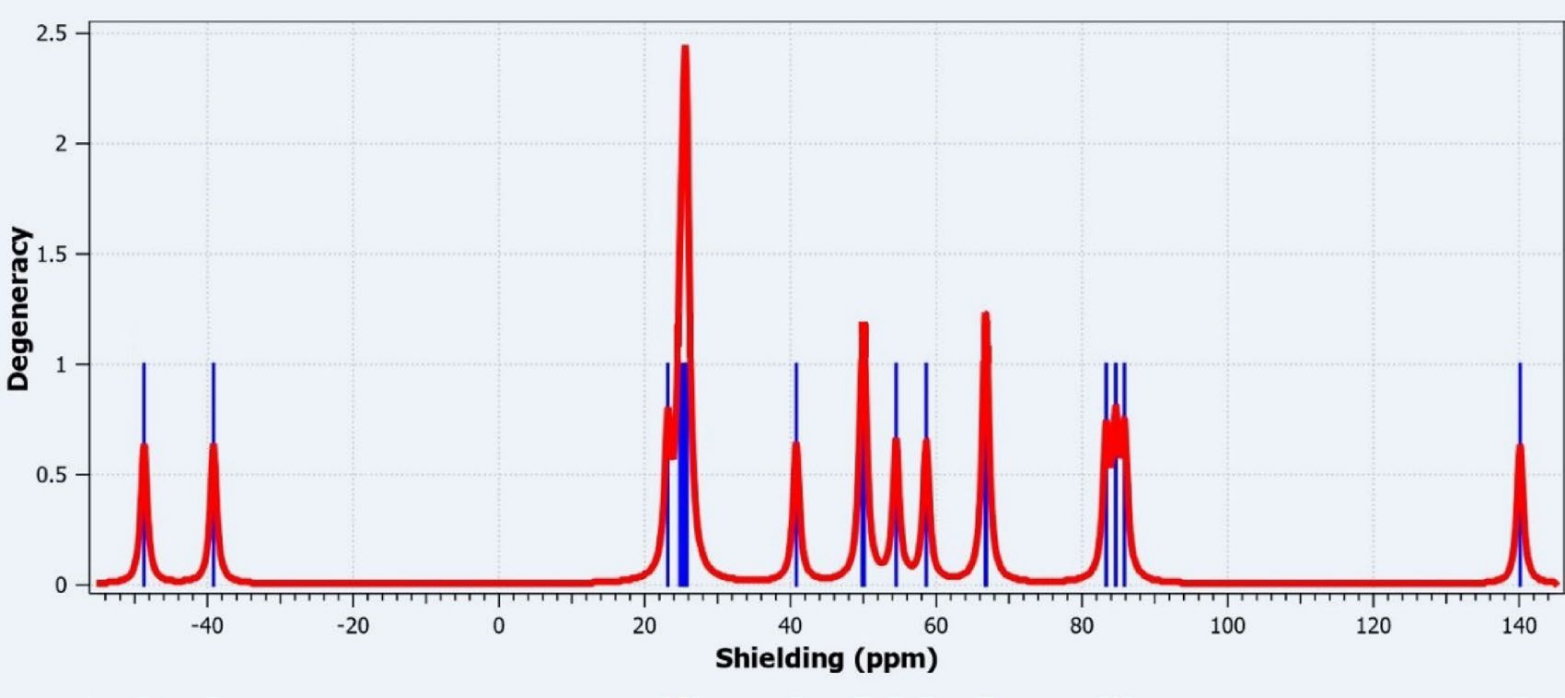
NMR spectrum of 3-(2-furyl)-1 H-pyrazole-5-carboxylic acid.

The NMR spectroscopy is a powerful analytical technique used in structural elucidation of compounds, as it enables the identification of hydrogen-containing groups and their neighboring environments^23^. Moreover, NMR is a highly valuable technique for understanding the relationship between molecular structure and electronic properties. By analyzing the behavior of nuclei in a magnetic field, distinct resonance signals can be obtained for each nucleus, providing critical insights into the skeletal framework of the compound^24^. The theoretical NMR spectrum of the 3-(2-furyl)-1 H-pyrazole-5-carboxylic acid compound is presented in Fig. 4. The calculations are based on DFT and include characteristic signals that reflect the chemical environment of each carbon atom. The horizontal axis represents the chemical shift values in parts per million (ppm), while the vertical axis indicates the intensity (degeneracy) of the signals. The red line corresponds to the theoretical total spectrum of all signals, whereas the blue lines represent the individual resonance signals associated with each carbon atom.

The signal observed around 140 ppm in the theoretical NMR spectrum corresponds to a carbon atom within the furan ring, which is directly bonded to an electronegative oxygen atom. While this carbon is often associated with aromatic-like behavior due to its involvement in a delocalized π-system, it is more precise to describe it as part of a conjugated heterocyclic system rather than truly aromatic in the classical sense. High δ value is attributed to the deshielding effect caused by the adjacent oxygen atom and the extended π-electron delocalization within the five-membered furan ring. This environment induces substantial electron withdrawal from the carbon nucleus, resulting in a resonance signal in the higher chemical shift region. The signals observed at approximately 80 ppm and in the ~ 60–70 ppm range are attributed to the conjugated aromatic carbons of the furan ring and the C–N bonded carbons within the pyrazole ring, respectively. Signals in the ~ 45–55 ppm range are likely associated with carbon atoms located further from heteroatoms in the aromatic system, possibly representing the more neutral regions of the furan ring. The signals in the lower ppm region (-40 ppm) indicate a strong shielding effect, which may arise in theoretical calculations for carbon atoms positioned near electronegative atoms such as nitrogen and oxygen.

### Density of States (DOS)

**Fig. 5:**
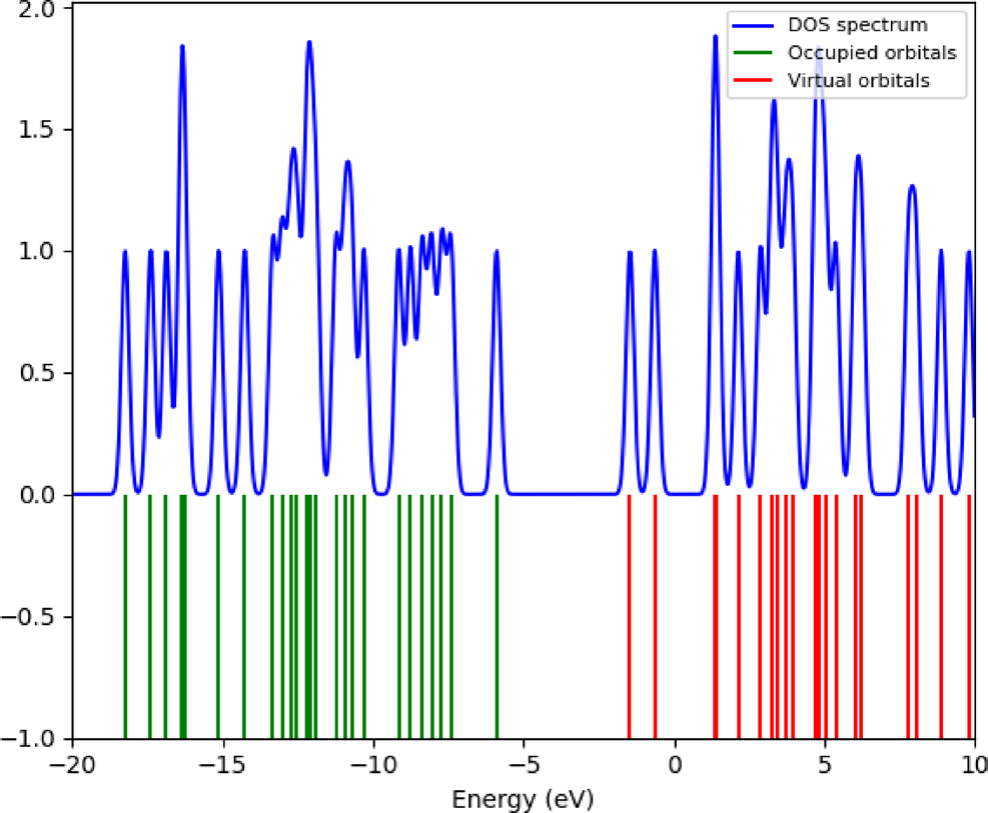
DOS of 3-(2-furyl)-1 H-pyrazole-5-carboxylic acid.

The DOS spectrum provides information on the compounds reactivity, stability, and optoelectronic potential by illustrating the density of electronic states at specific energy levels. In particular, it reveals the distribution of the compound’s energy levels and how the occupied and unoccupied molecular orbitals are concentrated with respect to energy^25^. DOS spectrum of the 3-(2-furyl)-1 H-pyrazole-5-carboxylic acid compound, calculated using DFT approach, is presented in Fig. 5. This spectrum was obtained using the B3LYP functional and the 6-31G(d) basis set within the framework of DFT.

The DOS spectrum presented in Fig. 5 illustrates the energy-dependent distribution of electronic states for the 3-(2-furyl)-1 H-pyrazole-5-carboxylic acid compound. The horizontal axis represents the energy values in electronvolts (eV), while the vertical axis indicates the DOS at each corresponding energy level. In this representation, the green lines denote occupied molecular orbitals, the red lines indicate unoccupied (virtual) orbitals, and the blue curve represents the total density of states (TDOS) profile. The occupied states, located in the negative energy region, reflect the electron configuration of the compound in its ground state and include the HOMO level. In contrast, the unoccupied states in the positive energy region begin at the LUMO level and correspond to potential energy levels involved in electronic excitations.

The notable width of the HOMO-LUMO energy gap observed in the spectrum provides significant insights into the compound’s electronic stability and chemical reactivity, suggesting that the molecule exhibits low reactivity and high electronic stability. Additionally, the sharp peaks observed in the DOS curve at certain energy levels indicate regions of high electronic density, which are attributed to the presence of conjugated π-systems. These features imply that the compound may be a promising candidate for advanced electronic and photophysical studies and could play a critical role in its optical absorption behavior.

## UV–Visible analysis

**Fig. 6:**
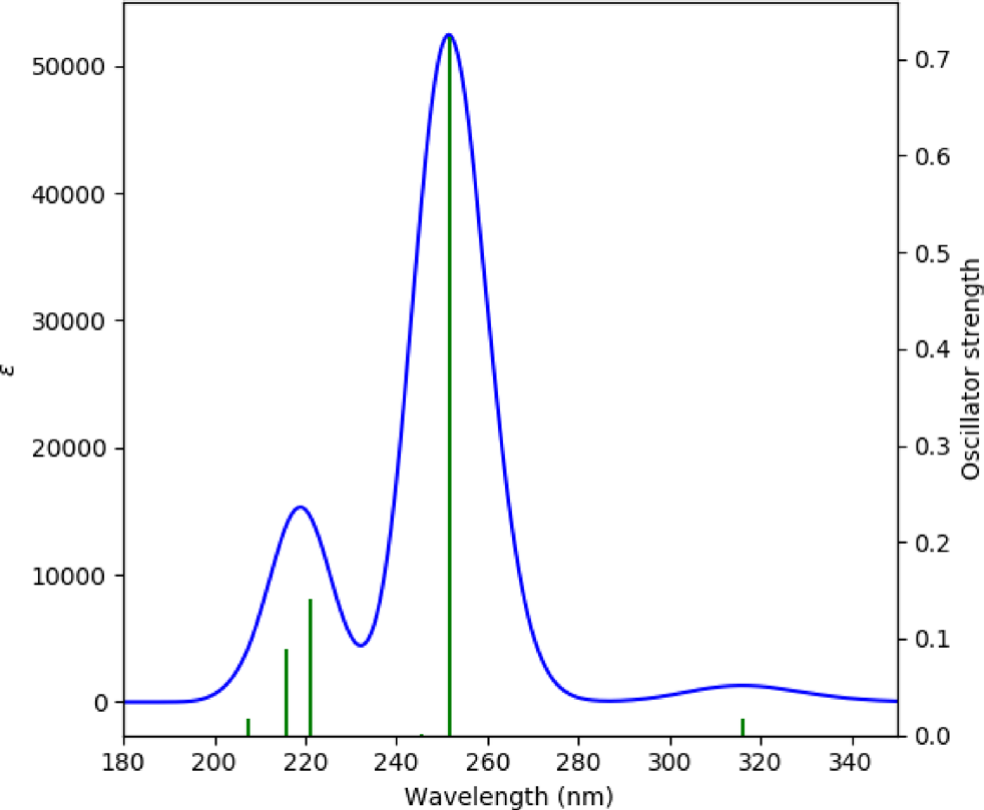
UV-visible absorption of 3-(2-furyl)-1 H-pyrazole-5-carboxylic acid.

To investigate the electronic transition characteristics of the 3-(2-furyl)-1 H-pyrazole-5-carboxylic acid molecule, a theoretical UV–Vis absorption spectrum was simulated using TD-DFT. These calculations were carried out at the B3LYP/6-31G(d) level of theory, based on the previously optimized molecular geometry. The theoretical UV-Vis spectrum is presented in Fig. 6.

As shown in Fig. 6, the horizontal axis represents the wavelength (nm), the left vertical axis indicates the molar absorptivity ε (L·mol^− 1^·cm^− 1^), and the right vertical axis corresponds to the oscillator strength (f). A prominent absorption peak is observed at approximately 251 nm, which has been explicitly assigned based on TD-DFT calculations as a π → π* transition involving the HOMO→LUMO + 1 excitation. This assignment was not inferred solely from the frontier molecular orbitals but was directly obtained from the TD-DFT excited-state calculations, providing a more robust and reliable interpretation of the optical transitions. Due to the π-conjugated aromatic structure of the compound, the spectrum exhibits distinct electronic transitions in the ultraviolet region.

The high molar absorptivity (~ 52,000) at ~ 251 nm suggests that this transition can be readily detected using UV-Vis spectrophotometry. Additionally, secondary peaks are observed in the range of 215–221 nm, with moderate oscillator strengths (0.08–0.14), indicating relevant transitions that contribute to the compound’s photophysical properties. A weak peak observed on the right side of the spectrum corresponds to a low-energy n→π* transition. The low oscillator strength (f < 0.02) suggests that the transition is either symmetry-forbidden or occurs with limited interaction.

The aromatic and conjugated framework of the molecule, stabilized by the furyl and pyrazole rings in conjunction with the carboxylic acid group, underpins these optical properties. The high absorptivity in the UV region indicates potential applications of the compound in areas such as UV-detector-based chromatography, photoactive systems, biomarker development, or sensor design. The absence of a distinct absorption band in the visible region implies that the compound is likely colorless and exhibits absorption behavior specific to the ultraviolet region.

## Molecular electrostatic potential (MEP)

**Fig. 7:**
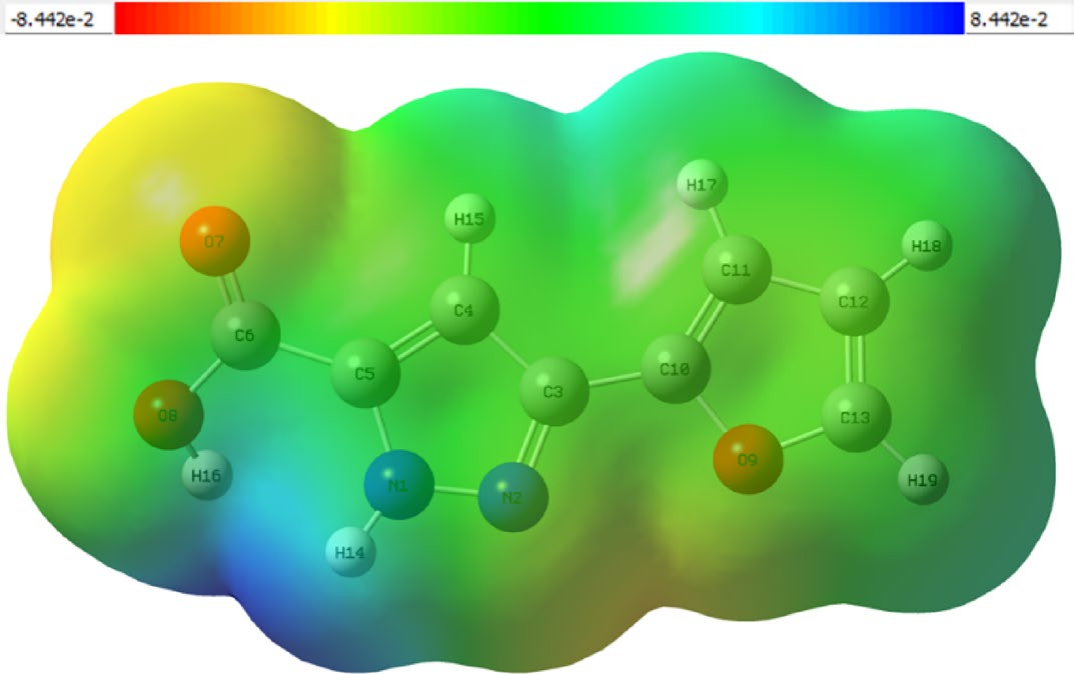
MEP of 3-(2-furyl)-1 H-pyrazole-5-carboxylic acid.

MEP maps obtained by quantum chemical DFT calculations contribute greatly to the understanding of the surface properties of molecules^26^. MEP analysis is used to visualise the electrostatic environment of a molecule on a three-dimensional surface to determine the reactive centres and possible interaction areas of the molecule. The MEP map obtained in this study is shown in Fig. 7 and the electrostatic potential of the 3-(2-furyl)-1 H-pyrazole-5-carboxylic acid molecule on its surface is calculated from the electron density of the molecule and is usually presented with a colour scale mapped onto the Van der Waals surface.

The color scale used in the MEP map presented in Fig. 7 ranges from − 8.442 × 10^− 2^ a.u. to + 8.442 × 10^− 2^ a.u. Regions transitioning from red to orange represent negative potential areas. A pronounced red-orange coloration is observed around the O7 and O9 atoms, indicating that these regions exhibit high electron density and act as nucleophilic sites susceptible to electrophilic attacks. Similarly, the O8 hydroxyl oxygen atom displays nucleophilic potential characterized by orange-green hues. The electron density in these areas highlights potential hydrogen-bonding sites and regions favorable for complexation interactions. Green-colored regions represent neutral areas, indicating a balanced electron density distribution. Aromatic rings and most of the carbon atoms exhibit green tones, suggesting these regions are electrostatically neutral or balanced and typically play a secondary role in chemical reactions. Regions colored toward blue signify positive potential areas characterized by low electron density, making them electrophilic and susceptible to nucleophilic attacks. The surroundings of the nitrogen atoms N1 and N2, particularly N1, are represented in light blue shades. This positive potential character around N1 is important due to its proton-accepting behavior. It is clearly understood from the MEP analysis that oxygen atoms in the carboxylic acid group predominantly possess nucleophilic character, whereas the nitrogen centers have a more electrophilic nature.

## Conclusion

This DFT-based theoretical study has comprehensively elucidated the structural, electronic, spectroscopic, and electrostatic properties of the 3-(2-furyl)-1 H-pyrazole-5-carboxylic acid molecule. The optimized molecular geometry revealed a planar configuration stabilized by intramolecular hydrogen bonding and conjugated π-systems. The vibrational analysis via FT-IR confirmed the presence of key functional groups, including carboxylic acid, furyl, and pyrazole rings, with calculated spectra showing strong agreement with expected characteristic vibrational modes. Theoretical NMR calculations provided detailed insight into the electronic environments of carbon atoms, supporting the molecular architecture and enabling precise structural identification. The electronic structure analysis indicated a relatively large HOMO–LUMO energy gap of 4.458 eV, signifying high electronic stability and low intrinsic reactivity, while still allowing potential for specific electronic transitions. DOS and UV-Vis spectral analyses revealed strong π→π* transitions and high absorption in the UV region, suggesting that the compound could serve in photoactive and optoelectronic applications. Furthermore, the MEP map identified electrophilic and nucleophilic regions across the molecular surface, particularly around oxygen and nitrogen atoms, providing a predictive tool for understanding potential sites for molecular interactions, hydrogen bonding, or complexation.

The findings obtained in this study not only provide a comprehensive understanding of the structural and functional characteristics of the molecule but also highlight its potential applicability in several advanced fields. Specifically, the strong π→π* absorption observed in the UV region supports the suitability of the compound for integration into UV detector-based optoelectronic devices. Furthermore, the nucleophilic and electrophilic regions identified through MEP analysis designate the compound as a promising candidate for chemical sensor design. In addition, its planar conformation, high electronic stability, and biologically relevant heterocyclic framework suggest that it may serve as a valuable scaffold in future drug design research.

## Data Availability

The datasets used and/or analysed during the current study available from the corresponding author on reasonable request.
